# The Association of Sedentary Behaviour and Cognitive Function in People Without Dementia: A Coordinated Analysis Across Five Cohort Studies from COSMIC

**DOI:** 10.1007/s40279-019-01186-7

**Published:** 2019-09-16

**Authors:** Carlijn M. Maasakkers, Jurgen A. H. R. Claassen, Paul A. Gardiner, Marcel G. M. Olde Rikkert, Darren M. Lipnicki, Nikolaos Scarmeas, Efthimios Dardiotis, Mary Yannakoulia, Kaarin J. Anstey, Nicolas Cherbuin, Mary N. Haan, Shuzo Kumagai, Kenji Narazaki, Tao Chen, Tze Pin Ng, Qi Gao, Ma S. Z. Nyunt, John D. Crawford, Nicole A. Kochan, Steve R. Makkar, Perminder S. Sachdev, COSMIC Collaborators, Dick H. J. Thijssen, René J. F. Melis

**Affiliations:** 1 Department of Geriatrics/Radboud Alzheimer Center, Radboud Institute for Health Sciences, Radboud University Medical Center, Route 925, Postbus 9101, 6500 HB Nijmegen, The Netherlands; 2 Department of Geriatrics/Radboud Alzheimer Center, Donders Institute for Brain Cognition and Behaviour, Radboud University Medical Center, Nijmegen, The Netherlands; 3 Centre for Health Services Research, Faculty of Medicine, The University of Queensland, Brisbane, Australia; 4 Mater Research Institute, The University of Queensland, South Brisbane, QLD Australia; 5 Centre for Healthy Brain Ageing (CheBA), School of Psychiatry, University of New South Wales, Sydney, Australia; 6 Department of Neurology, Aiginition Hospital, Medical School Athens, Athens, Greece; 7 Department of Neurology, Gertrude H Sergievsky Center, Taub Institute for Research in Alzheimer’s Disease and the Aging Brain, Columbia University, New York, USA; 8 Department of Neurology, University Hospital of Larissa, University of Thessaly, Larissa, Greece; 9 Department of Nutrition and Dietetics, School of Health Sciences and Education, Harokopio University, Athens, Greece; 10 Centre for Research on Ageing, Health and Wellbeing, Australian National University, Canberra, Australia; 11 School of Psychology, University of New South Wales, Sydney, Australia; 12 Neuroscience Research Australia, Randwick, Australia; 13 Department of Epidemiology and Biostatistics, School of Medicine, University of California San Francisco, San Francisco, USA; 14 Center for Health Science and Counseling, Kyushu University, Kasuga, Fukuoka Japan; 15 Department of Socio-Environmental Studies, Faculty of Socio-Environmental Studies, Fukuoka Institute of Technology, Higashi-ku, Fukuoka, Japan; 16 Department of Psychological Medicine, National University of Singapore, Singapore, Singapore; 17 Dementia Collaborative Research Centre, University of New South Wales, Sydney, Australia; 18 Department of Physiology, Radboud Institute for Health Sciences, Radboud University Medical Center, Nijmegen, The Netherlands; 19 Research Institute for Sport and Exercise Science, Liverpool John Moores University, Liverpool, UK

## Abstract

**Background:**

Besides physical activity as a target for dementia prevention, sedentary behaviour is hypothesized to be a potential target in its own right. The rising number of persons with dementia and lack of any effective treatment highlight the urgency to better understand these modifiable risk factors. Therefore, we aimed to investigate whether higher levels of sedentary behaviour are associated with reduced global cognitive functioning and slower cognitive decline in older persons without dementia.

**Methods:**

We used five population cohorts from Greece, Australia, USA, Japan, and Singapore (HELIAD, PATH, SALSA, SGS, and SLAS2) from the Cohort Studies of Memory in an International Consortium. In a coordinated analysis, we assessed the relationship between sedentary behaviour and global cognitive function with the use of linear mixed growth model analysis (mean follow-up range of 2.0–8.1 years).

**Results:**

Baseline datasets combined 10,450 older adults without dementia with a mean age range between cohorts of 66.7–75.1 years. After adjusting for multiple covariates, no cross-sectional association between sedentary behaviour and cognition was found in four studies. One association was detected where more sedentary behaviour was cross-sectionally linked to higher cognition levels (SLAS2, *B* = 0.118 (0.075; 0.160), *P* < 0.001). Longitudinally, there were no associations between baseline sedentary behaviour and cognitive decline (*P* > 0.05).

**Conclusions:**

Overall, these results do not suggest an association between total sedentary time and lower global cognition in older persons without dementia at baseline or over time. We hypothesize that specific types of sedentary behaviour may differentially influence cognition which should be investigated further. For now, it is, however, too early to establish undifferentiated sedentary time as a potential effective target for minimizing cognitive decline in older adults without dementia.

**Electronic supplementary material:**

The online version of this article (10.1007/s40279-019-01186-7) contains supplementary material, which is available to authorized users.

## Key Points

**Table:** 

In this study, the total time older adults spend sitting was not associated with lower cognitive performance or decline.
We hypothesize that specific types of SB may have a different effect on cognition depending on what a person is doing while sitting.
The results do not support targeting total sedentary time as a factor to reduce cognitive decline in older adults, despite its effects on cardiovascular risk factors.

## Introduction

The rising number of persons with dementia and the lack of effective treatments highlight the urgency for modifiable risk factors to be better understood, as these might account for around 30% of the population risk for dementia and Alzheimer’s disease [1–3]. Physical activity (PA) is one of these factors that has been shown in previous research [4], including a systematic review with meta-analysis in older adults, to have beneficial effects on cognition [5]. Traditionally, physical inactivity is mainly conceptualized as a lack of exercise. However, recent work suggested that sedentary behaviour (SB; low-intensity activities with a Metabolic Equivalent of Task (MET) of < 1.5) [6], which can be regarded as the other key component of physical inactivity, might be a target in its own right, due to its strong association with cardiovascular disease risk [7, 8]. Whilst most research focused on PA examined moderate-to-vigorous intensity PAs (MVPA; activities with a MET of ≥ 3.0, different from light PA which are activities with a MET of 1.5–2.9 [6]), older adults only spend 0.2 h per day on such activities, whereas SB is more prevalent in older adults with an average of 9.2 h per day [9–11]. Moreover, the Western population in general is becoming more sedentary, and physical inactivity represents the leading cause for all-cause mortality worldwide [12–14], making SB a potentially very important modifiable risk factor for dementia to target.

Several studies found aspects of SB to be associated, mostly after long-term, to various cardiovascular risk factors [15–19] and diseases [20]. Since these cardiovascular risk factors are associated with dementia as well, it raises the question of whether SB might also have an effect on cognition. In their review, Voss et al. endorse the hypothesis of an inverse relationship between SB and cognition, while acknowledging that conclusive research is currently lacking [21]. Besides these cardiovascular risk factors, that might underlie an association between SB and cognitive decline, also acute effects of SB related to vascular health and cerebral blood flow might be involved [22]. However, to date, experimental mechanistic studies on these acute effects of SB on cognition show conflicting findings [23–25]. For example, in an experiment with 9 overweight adults, reduced cognitive performance was seen after 6 h of uninterrupted sitting compared to intermittent bouts of standing [25]. In contrast, no beneficial effects were found on cognitive performance by either interrupting an 8-h sitting period among 6 healthy males [24], or 7-h sitting period among 19 overweight adults [23]. In their review of observational studies examining the long-term effects of SB on cognition, Falck et al. found associations between higher SB levels and lower cognition levels in six of the eight studies they reviewed [26]. In these studies, 8 different SB measures and 13 different cognitive measures were used [26]. This diversity in measurement types, together with other factors such as diverse study designs, different types of confounders corrected for, and small sample sizes previously mentioned, may have contributed to these contradictory results. This complexity emphasizes the need to further explore this association before SB can be regarded as a potential target to reduce the risk on cognitive impairment.

For this purpose, we performed a coordinated analysis examining the association between SB and cognition, which combined five different cohort datasets across the world. This enabled us to apply a comparable analysis model across large, compatible datasets, while consistently controlling for confounding variables thus enabling the interpretation of effects on a similar outcome measure. Specifically, we evaluated whether there was a cross-sectional relationship between higher baseline SB (duration of sitting per day) and lower baseline global cognitive function (Mini-Mental State Examination (MMSE) or Modified Mini-Mental State (3MS) Examination) in older adults without dementia, and whether baseline SB was related to prospective decline in cognitive function.

## Methods

### Datasets and Participants

Five studies in The Cohort Studies of Memory in an International Consortium (COSMIC) included a measure of SB and were, therefore, used in this investigation [27]. The first wave at which SB measures were collected was considered as the baseline wave for that study (i.e. SB was available at wave 1 for all studies except one (PATH study), where SB data were collected at wave 4). Information about the individual studies, including study abbreviations, can be found in the referenced literature (Table 1). People with cognitive impairment or dementia at baseline were excluded. This was based on self-reported (SGS cohort), algorithmic (PATH cohort) or clinical diagnosis based on the DSM-IV or DSM-V criteria (HELIAD, SALSA, and SLAS2 cohort). The algorithmic dementia diagnosis, used due to an absence of a clinical or self-reported diagnosis, was defined as a decline in MMSE scores between follow-up wave 3 and 4 of more than 2 standard deviations (SDs) or an MMSE of < 24 [28, 29]. All datasets were cleaned based on frequency tables, extreme values, and cross-checks in collaboration with the individual study teams.

**Table 1:** Cohort study information

Study	Hellenic Longitudinal Investigation of Aging and Diet	Personality and Total Health Through Life Project	Sacramento Area Latino Study on Aging	Sasaguri Genkimon Study	Singapore Longitudinal Ageing Studies (II)
Abbreviation	HELIAD	PATH	SALSA	SGS	SLAS2
Location	Larissa, Greece	Canberra, Australia	Sacramento, USA	Sasaguri, Japan	Singapore, Singapore
Waves used	W1–W2	W4	W1–W7	W1–W2	W1–W2
Length of follow-up	2.7 years	–	8.1 years	2.0 years	3.8 years
Age criteria	65 +	72 +	60 +	65 +	55 +
Dementia diagnosis	Clinical diagnosis	–	Clinical diagnosis	Self-reported	Clinical diagnosis
MCI/CIND diagnosis	Clinical MCI diagnosis	–	Clinical CIND diagnosis	–	Clinical MCI diagnosis
SB measure	Self-reported TV time	Self-reported sitting time on week/weekend day	Self-reported sitting time at work/at home/while driving a car	Objective accelerometer data	Self-reported sitting time on week/weekend day
Cognitive measure	MMSE (0–30)	MMSE (0–30)	3MS (0–100)	MMSE (0–30)	MMSE (0–30)
Starting year	2011	2001	1998	2011	2010
Reference	[30]	[31]	[32]	[33]	[34]

*MCI* Mild Cognitive Impairment, *CIND* cognitively impaired but not demented, *MMSE* Mini-Mental State Examination, *3MS* Modified Mini-Mental State

### Sedentary Behaviour, Cognition, and Covariates

Sedentary behaviour was measured in different ways across the five studies. Only one study (SGS) used an objective measure of SB, measured by a tri-axial accelerometer (Active style Pro HJA-350IT, Omron Healthcare, Kyoto, Japan) and transformed with an algorithm based on MET-scores into the fraction of sedentary time of total awake wear time [35]. One study (HELIAD) used a specific question that was part of the Athens Physical Activity Questionnaire which asked “During the last 7 days how many hours per day did you watch TV/video?” [36]. In two studies (PATH and SLAS2) participants were asked two questions relating to SB on a usual day, which distinguished between weekdays and weekend days. One study (SALSA) administered three questions of SB related to sitting at work, at home, and while driving a car during a regular week [37]. For each of the four studies which utilized self-reported measures of SB, a single SB variable was derived and transformed to total hours of sitting time per day (e.g. summing variables and dividing by seven).

Global cognition was measured with the MMSE in four of the studies (HELIAD, PATH, SGS, SLAS2). MMSE scores range from 0 to 30, with higher scores indicating better cognitive function. One study (SALSA) used the 3MS Examination, which measures the same concept but results in scores ranging from 0 to 100 [38].

Multiple variables were regarded as potential confounders of the relationship between SB and cognition, and were, therefore, included in the model including age, gender, ethnicity, education, income, BMI, morbidity count, perceived health, alcohol consumption, smoking status, marital status, living status, depression, sleep quality, blood pressure, and PA. Not all cohort studies had information on all covariates, and they were differently operationalised in the different studies. For PA, only MVPA was taken into account and was operationalised as hours per day of moderate-to-vigorous PA (PATH and SLAS2), MET hours (HELIAD and SALSA), or the fraction of moderate to vigorous PA of total awake wear time (SGS). Details regarding how the other covariates were operationalised across the different cohorts can be found in Table S1.

### Statistical Analysis

To deal with missing data, baseline values of all predictors (SB and confounders) were imputed using a multivariate imputation model (rates of missing data were 13.2% for HELIAD, 35.8% for PATH, 16.2% for SALSA, 33.4% for SGS, and 12.0% for SLAS2). An imputation model was used including all predictors and baseline outcome variables, with the fully conditional specification methods using predictive mean matching for all continuous variables (imputed datasets = 25, iterations = 100). Secondary analysis entailed a complete case analysis, including only cases which had information on all predictors (including SB) that were included in the model.

A coordinated analysis was used [39], with linear mixed growth models. Two adjusted models were used with the confounders stated above, one with and one without PA as a confounder since multicollinearity between PA and SB might be expected [40]. All models included baseline values of the predictors, and their interaction with linear time. Follow-up time was based on mean follow-up per wave per study, including two waves for three studies (HELIAD, SGS, and SLAS2), and seven for one study (SALSA). Stratified analyses for gender and PA based on median MVPA per study were performed.

To evaluate our model and outcome measures used, we assessed associations between the MMSE and a priori chosen factors known to impact MMSE (e.g. age, education, and depression) [41, 42].

Additionally, correlations between SB and each of the covariates were calculated. To evaluate the SB measures used in the context of our study, we assessed correlations between SB and a priori chosen factors known to be associated with SB (e.g. BMI, age, and morbidity count) [11, 43, 44]. Furthermore, we assessed the SB measures for ceiling and floor effects, and examined the medians and ranges. Analyses were performed, with two-sided testing with *P* values less than 0.05 considered significant, using SAS statistical software version 9.4.

## Results

Together the imputed datasets included 10,450 participants. A flowchart of the complete cases can be seen in Figure S1. Table 2 shows the baseline characteristics of the participants per study. Mean age within the cohorts ranged between 66.7 and 75.1 years, with a majority of female participants in most studies.

**Table 2:** Participant characteristics

Study	HELIAD	PATH	SALSA	SGS	SLAS2
Total *N*	1551	1552	1663	2597	3087
Age					
Mean (SD), years	72.5 (5.6)	75.1 (1.5)	70.2 (6.8)	73.4 (6.1)	66.7 (7.8)
QRange, years	69–76	74–76	55–74	68–78	61–72
Missing *N* (%)	7 (0.45)	1 (0.1)	0 (0.0)	0 (0.0)	0 (0.0)
Gender					
% female (N)	60.2 (933)	49.0 (760)	58.4 (971)	56.2 (1459)	62.6 (1932)
Missing *N* (%)	0 (0.0)	1 (0.1)	0 (0.0)	0 (0.0)	0 (0.0)
Years of education					
Mean (SD), years	7.8 (4.7)	14.4 (2.5)	7.3 (5.3)	11.1 (2.5)	5.8 (4.3)
Missing *N* (%)	1 (0.1)	4 (0.3)	0 (0.0)	23 (0.9)	56 (1.8)
BMI					
Mean (SD)	29.0 (4.7)	26.9 (5.0)	29.8 (6.0)	23.2 (3.2)	24.2 (4.1)
Missing *N* (%)	38 (2.5)	133 (8.6)	140 (8.4)	668 (25.7)	188 (6.1)
Morbidity count					
Mean (SD)	1.6 (1.2)	2.5 (1.5)	2.0 (1.8)	0.8 (0.8)	1.3 (1.2)
Missing *N* (%)	36 (2.3)	67 (4.3)	48 (2.9)	0 (0.0)	0 (0.0)
Sedentary behaviour					
Mean (SD)	3.5 (2.1) hours TV per day	7.1 (2.7) hours sitting per day	4.6 (2.3) hours sitting per day at home/work/driving a car	7.4 (2.1) hours SB of complete awake wear time^a^	6.1 (2.3) hours sitting per day
Missing *N* (%)	65 (4.2)	22 (1.4)	59 (3.6)	648 (25.0)	225 (7.3)
Moderate-to-vigorous Physical Activity					
Mean (SD)	1.2 (1.7) MET hours per day	0.5 (0.8) hours per day	10.2 (10.9) MET hours per day	0.7 (0.6) hours of complete awake wear time^a^	5.6 (2.3) hours per day
Missing *N* (%)	61 (3.9)	175 (11.3)	59 (3.6)	648 (25.0)	224 (7.3)
MMSE/3MS					
Mean (SD)	27.4/30 (2.3)	29.1/30 (1.1)	85.9/100 (11.6)	26.8/30 (2.7)	27.9/30 (2.6)
Missing *N* (%)	129 (8.3)	114 (7.4)	0 (0.0)	502 (19.3)	42 (1.4)

*BMI* body mass index, SB sedentary behaviour, *MMSE* Mini-Mental State Examination, *3MS* Modified Mini-Mental State, *QRange* interquartile range

^a^Based on mean (SD) total awake wear time of 13.6 (1.8) h

Unadjusted analysis (Table 3) showed no cross-sectional associations between SB and cognition in three studies (PATH, SGS, SLAS2). In one study (HELIAD), higher levels of SB were associated with lower MMSE scores, where 1 h/day of TV watching was associated with a 0.121 lower MMSE score (*B* = − 0.121, *P* < 0.001). In contrast, data from another study (SALSA) showed an increase in 3MS levels of 0.330 with every extra hour of SB/day (*B* = 0.330, *P* = 0.03). When the sub-questions in the SALSA study were analysed separately higher cognition levels were associated with lower levels of sitting at home (*B* = − 0.080, *P* = 0.005), while it was associated with higher levels of sitting while driving (*B* = 0.298, *P* < 0.001, see Table S3) in the unadjusted model. Adjusting for confounders resulted in point estimates suggesting smaller effects and loss of significance of the association between SB and cognition in both studies (HELIAD and SALSA). A positive cross-sectional association in one study (SLAS2) became significant after controlling for confounders, suggesting that every extra hour of SB/day was associated with a 0.118 higher cognition score (*B* = 0.118, *P* < 0.001). Stratified analysis by MVPA, however, showed that this was stronger for the high-PA group (Table S4). The stratified results in the SALSA study showed that higher SB levels were associated with worse cognition scores for the low-PA group, and with better cognition scores for the high-PA group. Stratification showed different unadjusted cross-sectional associations of SB with cognition by gender in SALSA, SGS, SLAS2 (Table S5).

**Table 3:** Linear mixed growth model analysis on the association of sedentary behaviour on cognition based on multiple imputed datasets

	Unadjusted			Model 1^a^			Model 2^b^		
	*B*	95% CI	*P* value	*B*	95% CI	*P* value	*B*	95% CI	*P* value
Cross-sectional effect									
HELIAD	− 0.121	− 0.190; − 0.052	< 0.001	− 0.028	− 0.091; 0.036	0.40	− 0.028	− 0.092; 0.036	0.40
PATH	− 0.003	− 0.005; 0.001	0.79	0.003	− 0.019; 0.024	0.81	0.001	− 0.021; 0.022	0.96
SALSA^c^	0.330	0.027; 0.632	0.03	− 0.070	− 0.341; 0.201	0.61	− 0.043	− 0.317; 0.230	0.76
SGS	− 0.005	− 0.015; 0.004	0.25	0.001	− 0.009; 0.011	0.80	0.006	− 0.006; 0.018	0.35
SLAS2	0.040	− 0.004; 0.083	0.08	0.062	0.023; 0.101	0.002	0.118	0.075; 0.160	< 0.001
Longitudinal effect									
HELIAD	0.030	− 0.020; 0.081	0.24	0.028	− 0.021; 0.077	0.26	0.028	− 0.021; 0.077	0.26
SALSA^c^	0.008	− 0.038; 0.053	0.74	− 0.006	− 0.053; 0.041	0.80	− 0.011	− 0.058; 0.037	0.66
SGS	− 0.003	− 0.009; 0.004	0.40	− 0.001	− 0.008; 0.006	0.75	− 0.001	− 0.010; 0.007	0.73
SLAS2	− 0.007	− 0.021; 0.007	0.32	− 0.011	− 0.025; 0.003	0.12	− 0.011	− 0.027; 0.004	0.16

The basic linear mixed model (ignoring covariate adjustment) was parameterized as: cognition (MMSE or 3MS) = *x*_1_ + *x*_2_ × sedentary behaviour + *x*_3_ × time + *x*_4_ × time × sedentary behaviour + random intercept for each individual + residual error. The cross-sectional effects presented here are then represented in this model as *x*_2_ and the longitudinal effect is *x*_4_

^a^Model 1 is adjusted for age, gender, ethnicity, education, income, alcohol consumption, smoking, BMI, marital status, living status, perceived health, morbidities, blood pressure, sleep quality, and depression. In HELIAD not corrected for ethnicity, income, perceived health. In PATH not corrected for ethnicity. In SLAS2 not corrected for income. In SGS not corrected for ethnicity, marital status, blood pressure, sleep quality

^b^Model 2 is adjusted for all variables of model 1 + PA

^c^SALSA outcome variable is 3MS ranging from 0 to 100 instead of MMSE ranging from 0 to 30

Over a mean follow-up time of 2.7, 8.1, 2.0, and 3.8 years, cognitive function (SD) changed by − 0.94 (3.53, *P* < 0.001), − 7.56 (16.79, *P* < 0.001), 0.10 (2.48, *P* = 0.22), and − 0.49 (1.98, *P* < 0.001) for the studies HELIAD, SALSA, SGS, and SLAS2, respectively. No associations between SB and decline in MMSE/3MS were found in both the unadjusted and adjusted models. However, the stratified results from the longitudinal analysis showed positive associations between SB and MMSE in one study (HELIAD) in the low-PA group only (*B* = 0.072, *P* = 0.04), see Table S4. For the stratified analysis by gender, no differences were seen (Table S5). The secondary complete case analysis showed the same results (see Table S2) as the primary analysis using the imputed datasets.

**Fig. 1:**
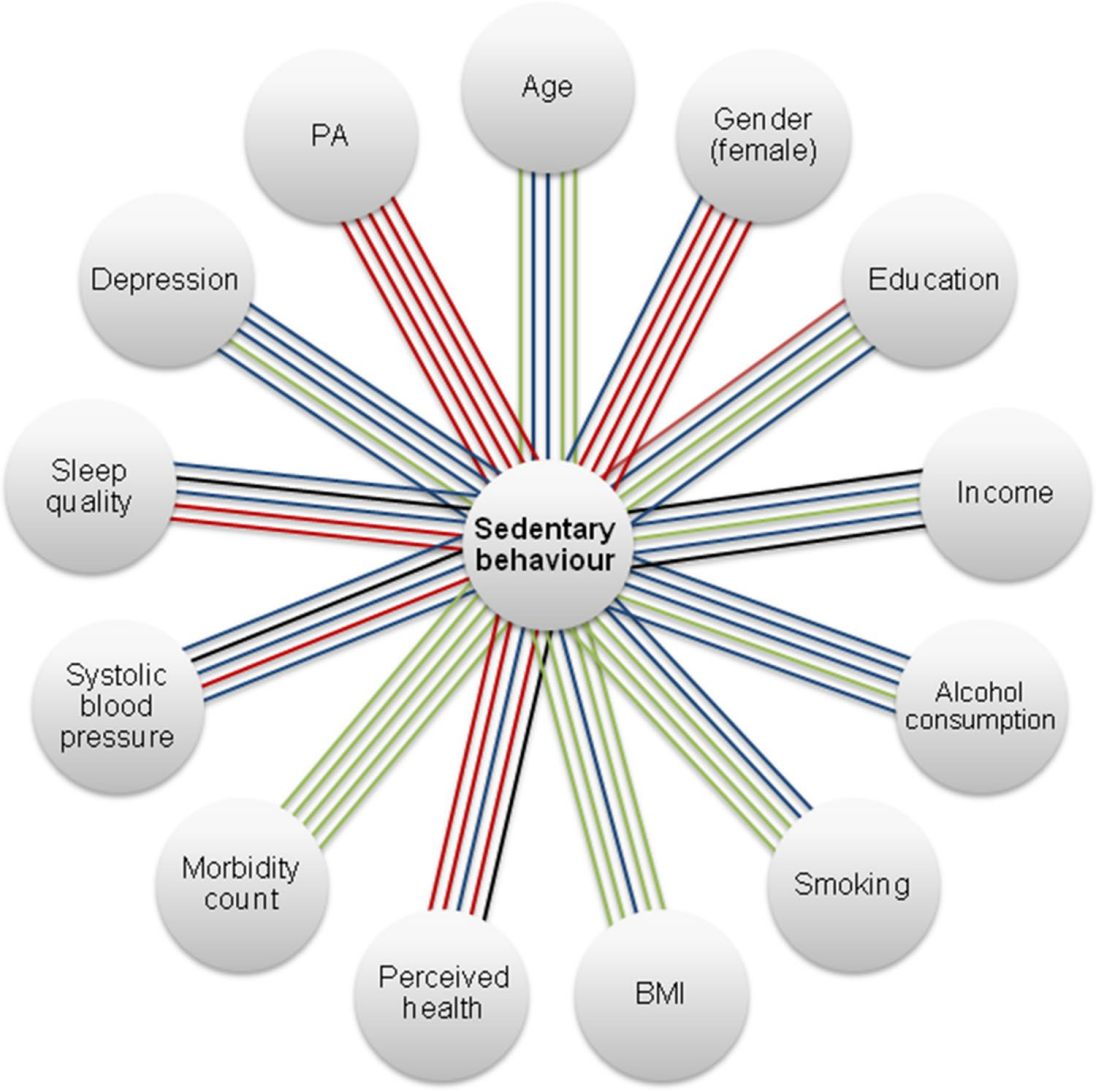
Correlation analysis of sedentary behaviour measures with imputed covariates. Lines represent studies, in similar order for each covariate seen from the middle (1 = HELIAD, 2 = PATH, 3 = SALSA, 4 = SGS, 5 = SLAS2). Green lines represent positive significant associations (high SB, high covariate). Red lines represent inverse significant associations (high SB, low covariate). Blue lines represent non-significant associations (SB not related to covariate). Black lines indicate that the covariate was not measured in that particular study

Correlations between SB and covariates can be seen per study in Fig. 1. Inverse associations were found between SB and PA in all studies (three weak, two moderate associations, see Table S6). The evaluation of the model can be found in the S1 Results.

## Discussion

This is the first study reporting on the association between SB and cognition in multiple population-based cohorts combining large samples of individual participant data. The coordinated analysis design enabled us to preserve detailed information on all main variables and covariates, as well as to test the replicability of the results across studies [45]. Longitudinally, no associations between SB and the rate of cognitive decline were found. This was unexpected given recent studies reported short-term and long-term detrimental effects of SB on the brain [25, 46], although other studies could not establish an association between SB and cognition [24, 47].

What can explain the observed absence of a relationship between SB and cognition found in this coordinated analysis of five cohort studies? First of all, in our study, SB was operationalised as total sedentary time, as commonly done in this field of research. However, based on the current findings, we hypothesize the type of SB is important. As a result, it is possible that considering them together might have limited our capacity to detect their individual effects, since it could be that only specific types of SB have a negative influence on cognition. Take, for example, TV watching for which a cross-sectional association was seen in our unadjusted HELIAD results and previous studies [21, 48, 49]. TV viewing may require lower cognitive engagement than sitting while reading or making puzzles. In turn, different cognitive engagement is likely to be associated with differences in cerebral blood flow and oxygenated haemoglobin levels [50, 51]. We, therefore, speculate that the type of mental activity engaged in while seated moderates the effect SB has on the brain and cognition. This may also explain why in one study (SALSA), the results of the unadjusted cross-sectional analysis revealed a positive association, while when the cross-sectional relations were analysed separately sitting while driving was associated with higher cognition levels compared to sitting at home which was associated with lower cognition levels. Additionally, there are indications that it is not the total time of SB that is of influence but rather the extent to which a sitting bout is broken up by periods of standing or walking [52]. All in all, this suggests that potential effects of SB on the brain work via more specific ways than via total sitting time. The operationalisation of SB as total undifferentiated sedentary time might, therefore, in retrospect have resulted in the lack of a clear association. It is, therefore, necessary in future studies in this area to assess SB with multiple domain-specific questions on types of behaviour, reducing the risk of underestimation and improving validity [53].

The measures of our primary independent variable, SB, that were used in this study need to be critically assessed, as previous studies pointed to the potential problems with validity of SB questionnaires, and poor correlation of for example TV-time with total sitting time [7, 54, 55]. Especially single-item questions are known to result in an underestimation of total sitting time [56]. Additionally, since cognitive function is a factor that explains discrepancies between objective and subjective activity measures, recall bias makes subjective assessments in the older population less reliable [57]. Although this population concerned participants without cognitive impairment at baseline, we cannot rule out an information bias, which highlights the fact that research regarding SB is still in its infancy. For these reasons, we evaluated the associations of the SB measures with measures of BMI, age, and morbidity count, that were expected to result in positive significant correlations [11, 43, 44]. The expected correlations with SB, although small, were indeed verified in most studies. Furthermore, the lack of floor effects of the measures used indicate the SB measures had the capacity to discriminate between varying levels of SB, further reinforcing that the SB measures retained sufficient study-specific validity to serve as a marker of total SB. However, domain-specific assessment of SB is likely to both improve the assessment of total SB as well as add the possibility of studying outcomes in relation to different types of SB.

The same critical approach should be used to assess the study-specific validity of the outcome measures used. Although commonly used, the MMSE is criticized for not being sensitive enough to distinguish deterioration [58], and is affected by strong ceiling effects [59]. Especially in adults without cognitive impairment, it may not be sensitive to individual differences. However, the evaluation we performed on our model showed the analytical approach was sensitive enough to detect the association between known (risk) factors and MMSE scores [41, 42]. For age, education, and depression, significant associations were found in all of the five cohort studies in the adjusted models. This strengthens the validity of the model used in our study and lends further support to the rigour of our null findings for the association of SB and global cognition. Future research should nonetheless examine associations between SB and performance on specific cognitive domains [48]. Based on the improvements seen in executive functioning measures after aerobic training [60], this domain might be particularly prone to the negative effects of SB.

Moreover, the lack of an association between SB and cognition found in our study could be because we adjusted more adequately for confounding compared to some of the earlier studies. Previous research also reported that significant associations dissipated after correcting for confounders [47]. The existing literature and the correlations we observed between SB and each of the covariates (Fig. 1) show that multiple variables influence both SB and cognition, and may thus confound the association between SB and cognition. This can even differ for the different types of SB. TV viewing is known, for example, to be related to high caloric intake [61, 62], and the degree of social engagement can differ between varying SBs. Potentially, certain risk factors thus cluster with different types of SBs. This clustering might be one of the underlying reasons relationships between SB and cognition can potentially vary depending on the type of SB. Due to the thorough way in which the confounders were selected, it is reasonable to assume that the majority of potential confounders were included in our analysis and that residual confounding is not significantly impacting our results. The inclusion of these covariates may have had the effect of attenuating associations between SB and cognition; however, no clear directional association between SB and cognition was seen in models where the covariates were not adjusted for either. However, related to confounders, performing stratified analyses on MVPA revealed different effects of SB for the low-PA group versus the high-PA group in two studies (SALSA and SLAS2). This effect modification by PA has previously been shown for the relation between SB and mortality [63]. Therefore, this suggests that MVPA can, to some extent, counteract a potential detrimental effect of SB on cognition, creating a need for more comprehensive studies examining both factors simultaneously. Stratification did reveal gender differences in the unadjusted cross-sectional associations of SB and cognition in some studies, but no pattern of effect modification by gender consistently in the same direction could be replicated across studies.

Lastly, it is possible that historical SB (either accumulated SB or SB during another life phase) is of greater influence on cognitive function in older adults compared to current SB as investigated here. A similar mechanism is seen for smoking, in which cumulative smoking in pack-years is of greater influence on current mortality risk [64]. Relatedly, there is a possibility that one’s current SB is not an accurate reflection of one’s history of SB. Particularly around the age of retirement, it is expected that the amount of SB changes due to a changing daily routine [65]. This, in combination with the relative short time interval used for the longitudinal analysis, may have contributed to the lack of an association found between baseline SB and cognition decline. Therefore, while our findings diminish the likelihood of total undifferentiated sedentary time in late life as a risk factor for cognitive decline, the impact of cumulative or earlier life SB on cognitive decline remains to be elucidated. This would also give the opportunity to study the concept of reverse causation that could bias results.

The impact of the limitations scrutinized above should be balanced against the strengths of the current study. In addition to the aforementioned rigour of the confounder selection and the evaluation we performed to show the study specific validity of both the measures of total SB and global cognition, the main strength lies in the applied coordinated analysis approach. High-quality cohort study data were used that were made available through the COSMIC initiative. The coordinated analysis approach allowed for replication across the five data sets using a comparable analysis model and adds further rigour to the null finding for the association between total SB and global cognition. Finally, the generalizability of our findings benefitted strongly from the population-based character of the cohorts included and their geographical spread across the world.

### Practical Implications

In this study, total undifferentiated sedentary time in late life was not significantly associated with lower global cognitive performance or decline. Given the many other deleterious consequences of prolonged sitting on one’s health, guidelines recommending reductions in sitting time are still relevant. However, we did not find any evidence to establish total sedentary time as a targetable risk factor in the prevention strategy for dementia. This finding should be seen as a guide mark instead of an end-point, and as such offer important suggestions for the best way to move this field forward. The limitations highlighted in our study are inherent to the current state of this field of research. Therefore, we hope these notions can be used to investigate the relationship between SB and cognition in a more optimal way. Specifically, we propose a shift of attention for future studies in not only looking at total SB but more task-specific SBs using validated questionnaires that are able to measure types of SB separately and bring this in relation to more sensitive cognitive outcome measures. Also more insight needs to be gained into the physiological mechanisms by which SB potentially influences brain health and cognition. To do so, we need to go beyond epidemiological studies alone, and combine epidemiological analyses with clinical experiments that focus on the mechanistic effects of SB on the brain and cognition. In this way, we will be better able to understand if SB is a potential target for cognitive decline or not.

## Conclusions

Across the five population cohorts examined, this study did not find support for an association between total undifferentiated sedentary time and lower global cognition, at baseline or over time. We hypothesize that specific types of SB may differentially influence cognition depending on what a person is doing while sitting. Future research should investigate this further using sensitive neuropsychological tests and investigating mechanisms underlying the potential relationship between SB and cognition. For now, it is, however, too early to establish undifferentiated sedentary time as a potential preventable risk-factor for dementia prevention.

## Electronic supplementary material

Below is the link to the electronic supplementary material.

Supplementary material 1 (DOCX 151 kb)

## Data Availability

Data cannot be shared openly based on the terms of each study; however, researchers may apply to the COSMIC Scientific Steering Committee by contacting Dr. Kristan Kang (k.kang@unsw.edu.au).

## Abbreviations

3MS: Modified Mini-Mental State Examination
CIND: Cognitively impaired but not demented
COSMIC: Cohort Studies of Memory in an International Consortium
HELIAD: Hellenic Longitudinal Investigation of Aging and Diet
MCI: Mild Cognitive Impairment
MET: Metabolic Equivalent of Task
MMSE: Mini-Mental State Examination
MVPA: Moderate-to-vigorous physical activity
PA: Physical activity
PATH: Personality and Total Health Through Life Project
SALSA: Sacramento Area Latino Study on Aging
SB: Sedentary behaviour
SGS: Sasaguri Genkimon Study
SLAS2: Singapore Longitudinal Ageing Studies (II)

## References

[1] ADI. World Alzheimer Report 2015. London; 2015.

[2] Norton S, Matthews FE, Barnes DE, Yaffe K, Brayne C (2014). Potential for primary prevention of Alzheimer’s disease: an analysis of population-based data. Lancet Neurol.

[3] de Bruijn RF, Bos MJ, Portegies ML, Hofman A, Franco OH, Koudstaal PJ (2015). The potential for prevention of dementia across two decades: the prospective, population-based Rotterdam Study. BMC Med.

[4] Kramer AF, Erickson KI, Colcombe SJ (2006). Exercise, cognition, and the aging brain. J Appl Physiol.

[5] Northey JM, Cherbuin N, Pumpa KL, Smee DJ, Rattray B (2018). Exercise interventions for cognitive function in adults older than 50: a systematic review with meta-analysis. Br J Sports Med.

[6] Gibbs BB, Hergenroeder AL, Katzmarzyk PT, Lee I-M, Jakicic JM (2015). Definition, measurement, and health risks associated with sedentary behavior. Med Sci Sports Exerc.

[7] Thyfault JP, Du M, Kraus WE, Levine JA, Booth FW (2015). Physiology of sedentary behavior and its relationship to health outcomes. Med Sci Sports Exerc.

[8] Carter S, Hartman Y, Holder S, Thijssen DH, Hopkins ND (2017). Sedentary behavior and cardiovascular disease risk: mediating mechanisms. Exerc Sport Sci Rev.

[9] Wheeler MJ, Dempsey PC, Grace MS, Ellis KA, Gardiner PA, Green DJ (2017). Sedentary behavior as a risk factor for cognitive decline? A focus on the influence of glycemic control in brain health. Alzheimers Dement.

[10] Healy GN, Matthews CE, Dunstan DW, Winkler EAH, Owen N (2011). Sedentary time and cardio-metabolic biomarkers in US adults: NHANES 2003–06. Eur Heart J.

[11] Matthews CE, Chen KY, Freedson PS, Buchowski MS, Beech BM, Pate RR (2008). Amount of time spent in sedentary behaviors in the United States, 2003–2004. Am J Epidemiol.

[12] Kohl HW, Craig CL, Lambert EV, Inoue S, Alkandari JR, Leetongin G (2012). The pandemic of physical inactivity: global action for public health. Lancet.

[13] Lee IM, Shiroma EJ, Lobelo F, Puska P, Blair SN, Katzmarzyk PT (2012). Effect of physical inactivity on major non-communicable diseases worldwide: an analysis of burden of disease and life expectancy. Lancet.

[14] Owen N, Sparling PB, Healy GN, Dunstan DW, Matthews CE (2010). Sedentary behavior: emerging evidence for a new health risk. Mayo Clin Proc.

[15] Stamatakis E, Hamer M, Tilling K, Lawlor DA (2012). Sedentary time in relation to cardio-metabolic risk factors: differential associations for self-report vs accelerometry in working age adults. Int J Epidemiol.

[16] Henson J, Yates T, Biddle SJ, Edwardson CL, Khunti K, Wilmot EG (2013). Associations of objectively measured sedentary behaviour and physical activity with markers of cardiometabolic health. Diabetologia.

[17] Huynh QL, Blizzard CL, Sharman JE, Magnussen CG, Dwyer T, Venn AJ (2014). The cross-sectional association of sitting time with carotid artery stiffness in young adults. BMJ Open.

[18] Parsons TJ, Sartini C, Ellins EA, Halcox JP, Smith KE, Ash S (2016). Objectively measured physical activity and sedentary behaviour and ankle brachial index: Cross-sectional and longitudinal associations in older men. Atherosclerosis.

[19] Pinto Pereira SM, Power C (2013). Sedentary behaviours in mid-adulthood and subsequent body mass index. PLoS One.

[20] Beunza JJ, Martinez-Gonzalez MA, Ebrahim S, Bes-Rastrollo M, Nunez J, Martinez JA (2007). Sedentary behaviors and the risk of incident hypertension: the SUN Cohort. Am J Hypertens.

[21] Voss MW, Carr LJ, Clark R, Weng T (2014). Revenge of the “sit” II: Does lifestyle impact neuronal and cognitive health through distinct mechanisms associated with sedentary behavior and physical activity?. Ment Health Phys Act.

[22] Wheeler MJ, Dunstan DW, Smith B, Smith KJ, Scheer A, Lewis J (2019). Morning exercise mitigates the impact of prolonged sitting on cerebral blood flow in older adults. J Appl Physiol.

[23] Wennberg P, Boraxbekk C-J, Wheeler M, Howard B, Dempsey PC, Lambert G (2016). Acute effects of breaking up prolonged sitting on fatigue and cognition: a pilot study. BMJ Open.

[24] Vincent GE, Jay SM, Sargent C, Kovac K, Vandelanotte C, Ridgers ND (2017). The impact of breaking up prolonged sitting on glucose metabolism and cognitive function when sleep is restricted. Neurobiol Sleep Circadian Rhythms.

[25] Mullane SL, Buman MP, Zeigler ZS, Crespo NC, Gaesser GA (2017). Acute effects on cognitive performance following bouts of standing and light-intensity physical activity in a simulated workplace environment. J Sci Med Sport.

[26] Falck RS, Davis JC, Liu-Ambrose T (2017). What is the association between sedentary behaviour and cognitive function? A systematic review. Br J Sports Med.

[27] Sachdev PS, Lipnicki DM, Kochan NA, Crawford JD, Rockwood K, Xiao S (2013). COSMIC (Cohort Studies of Memory in an International Consortium): an international consortium to identify risk and protective factors and biomarkers of cognitive ageing and dementia in diverse ethnic and sociocultural groups. BMC Neurol.

[28] Ruitenberg A, den Heijer T, Bakker SL, van Swieten JC, Koudstaal PJ, Hofman A (2005). Cerebral hypoperfusion and clinical onset of dementia: the Rotterdam Study. Ann Neurol.

[29] Van den Kommer T, Comijs H, Dik M, Jonker C, Deeg D (2008). Development of classification models for early identification of persons at risk for persistent cognitive decline. J Neurol.

[30] Dardiotis E, Kosmidis MH, Yannakoulia M, Hadjigeorgiou GM, Scarmeas N (2014). The Hellenic Longitudinal Investigation of Aging and Diet (HELIAD): rationale, study design, and cohort description. Neuroepidemiology.

[31] Anstey KJ, Christensen H, Butterworth P, Easteal S, Mackinnon A, Jacomb T (2011). Cohort profile: the PATH through life project. Int J Epidemiol.

[32] Haan MN, Mungas DM, Gonzalez HM, Ortiz TA, Acharya A, Jagust WJ (2003). Prevalence of dementia in older Latinos: the influence of type 2 diabetes mellitus, stroke and genetic factors. J Am Geriatr Soc.

[33] Narazaki K, Nofuji Y, Honda T, Matsuo E, Yonemoto K, Kumagai S (2013). Normative data for the Montreal Cognitive Assessment in a Japanese community-dwelling older population. Neuroepidemiology.

[34] Ng TP, Camous X, Nyunt MSZ, Vasudev A, Tan CTY, Feng L (2015). Markers of T-cell senescence and physical frailty: insights from Singapore Longitudinal Ageing Studies. NPJ Aging Mech Dis.

[35] Chen T, Narazaki K, Honda T, Chen S, Haeuchi Y, Nofuji YY (2015). Tri-axial accelerometer-determined daily physical activity and sedentary behavior of suburban community-dwelling older Japanese adults. J Sports Sci Med.

[36] Kavouras S, Maraki M, Kollia M, Gioxari A, Jansen L, Sidossis L (2016). Development, reliability and validity of a physical activity questionnaire for estimating energy expenditure in Greek adults. Sci Sports.

[37] Shih I-F, Paul K, Haan M, Yu Y, Ritz B (2018). Physical activity modifies the influence of apolipoprotein E ε4 allele and type 2 diabetes on dementia and cognitive impairment among older Mexican Americans. Alzheimers Dement.

[38] Teng E, Chui H (1987). The modified mini-mental state examination (3MS). Can J Psychiatry.

[39] Hofer SM, Piccinin AM (2009). Integrative data analysis through coordination of measurement and analysis protocol across independent longitudinal studies. Psychol Methods.

[40] Talarico R, Janssen I (2018). Compositional associations of time spent in sleep, sedentary behavior and physical activity with obesity measures in children. Int J Obes.

[41] Crum RM, Anthony JC, Bassett SS, Folstein MF (1993). Population-based norms for the Mini-Mental State Examination by age and educational level. JAMA.

[42] Nebes RD, Butters M, Mulsant B, Pollock B, Zmuda M, Houck P (2000). Decreased working memory and processing speed mediate cognitive impairment in geriatric depression. Psychol Med.

[43] Rosenberg DE, Norman GJ, Wagner N, Patrick K, Calfas KJ, Sallis JF (2010). Reliability and validity of the Sedentary Behavior Questionnaire (SBQ) for adults. J Phys Act Health.

[44] Young DR, Hivert M-F, Alhassan S, Camhi SM, Ferguson JF, Katzmarzyk PT (2016). Sedentary behavior and cardiovascular morbidity and mortality: a science advisory from the American Heart Association. Circulation.

[45] Peng RD, Dominici F, Zeger SL (2006). Reproducible epidemiologic research. Am J Epidemiol.

[46] Vancampfort D, Stubbs B, Lara E, Vandenbulcke M, Swinnen N, Smith L (2018). Mild cognitive impairment and sedentary behavior: a multinational study. Exp Gerontol.

[47] Kesse-Guyot E, Andreeva VA, Lassale C, Hercberg S, Galan P (2014). Clustering of midlife lifestyle behaviors and subsequent cognitive function: a longitudinal study. Am J Public Health.

[48] Kesse-Guyot E, Charreire H, Andreeva VA, Touvier M, Hercberg S, Galan P (2012). Cross-sectional and longitudinal associations of different sedentary behaviors with cognitive performance in older adults. PLoS One.

[49] Hamer M, Stamatakis E (2014). Prospective study of sedentary behavior, risk of depression, and cognitive impairment. Med Sci Sports Exerc.

[50] Gur RC, Gur RE, Obrist WD, Hungerbuhler JP, Younkin D, Rosen AD (1982). Sex and handedness differences in cerebral blood flow during rest and cognitive activity. Science.

[51] Villringer A, Planck J, Hock C, Schleinkofer L, Dirnagl U (1993). Near infrared spectroscopy (NIRS): a new tool to study hemodynamic changes during activation of brain function in human adults. Neurosci Lett.

[52] Thosar SS, Bielko SL, Mather KJ, Johnston JD, Wallace JP (2015). Effect of prolonged sitting and breaks in sitting time on endothelial function. Med Sci Sports Exerc.

[53] Lyden K, Kozey Keadle SL, Staudenmayer JW, Freedson PS (2012). Validity of two wearable monitors to estimate breaks from sedentary time. Med Sci Sports Exerc.

[54] Healy GN, Clark BK, Winkler EA, Gardiner PA, Brown WJ, Matthews CE (2011). Measurement of adults’ sedentary time in population-based studies. Am J Prev Med.

[55] Stamatakis E, Ekelund U, Ding D, Hamer M, Bauman AE, Lee I-M (2019). Is the time right for quantitative public health guidelines on sitting? A narrative review of sedentary behaviour research paradigms and findings. Br J Sports Med.

[56] Clemes SA, David BM, Zhao Y, Han X, Brown W (2012). Validity of two self-report measures of sitting time. J Phys Act Health.

[57] Herbolsheimer F, Riepe MW, Peter R (2018). Cognitive function and the agreement between self-reported and accelerometer-accessed physical activity. BMC Geriatr.

[58] Mazzoni M, Ferroni L, Lombardi L, Del Torto E, Vista M, Moretti P (1992). Mini-Mental State Examination (MMSE): sensitivity in an Italian sample of patients with dementia. Ital J Neurol Sci.

[59] Franco-Marina F, García-González JJ, Wagner-Echeagaray F, Gallo J, Ugalde O, Sánchez-García S (2010). The Mini-mental State Examination revisited: ceiling and floor effects after score adjustment for educational level in an aging Mexican population. Int Psychogeriatr.

[60] Bherer L, Erickson KI, Liu-Ambrose T (2013). A review of the effects of physical activity and exercise on cognitive and brain functions in older adults. J Aging Res.

[61] Pardee PE, Norman GJ, Lustig RH, Preud’homme D, Schwimmer JB (2007). Television viewing and hypertension in obese children. Am J Prev Med.

[62] Williams DM, Raynor HA, Ciccolo JT (2008). A review of TV viewing and its association with health outcomes in adults. Am J Lifestyle Med.

[63] Stamatakis E, Gale J, Bauman A, Ekelund U, Hamer M, Ding D (2019). Sitting time, physical activity, and risk of mortality in adults. J Am Coll Cardiol.

[64] Liaw K-M, Chen C-J (1998). Mortality attributable to cigarette smoking in Taiwan: a 12-year follow-up study. Tob Control.

[65] Touvier M, Bertrais S, Charreire H, Vergnaud A-C, Hercberg S, Oppert J-M (2010). Changes in leisure-time physical activity and sedentary behaviour at retirement: a prospective study in middle-aged French subjects. Int J Behav Nutr Phys Act.

