# Supplementary material for: The Association of Sedentary Behaviour and Cognitive Function in People Without Dementia: A Coordinated Analysis Across Five Cohort Studies from COSMIC
Source: Sports Med. 2019 Sep 16;50(2):403–13. doi: 10.1007/s40279-019-01186-7 (PMC6985182; doi:10.1007/s40279-019-01186-7)
Supplement: Supplementary file 1 — Supplementary material 1 (DOCX 151 kb) [file 40279_2019_1186_MOESM1_ESM.docx]

Additional files

Table S1 – Covariate operationalisation

Figure S1 – Flow chart complete cases

Table S2 – Linear mixed growth model analysis for complete cases on the association of sedentary behaviour on cognition

Table S3 – Linear mixed model analysis on the cross-sectional association of different types of sedentary behaviour on cognition in SALSA

Table S4 – Linear mixed growth model analysis for imputed cases on the association of sedentary behaviour on cognition stratified by physical activity level

Table S5 – Linear mixed growth model analysis for imputed cases on the association of sedentary behaviour on cognition stratified by gender

Table S6 – Correlations of sedentary behaviour with imputed covariables

S1 Results – Model evaluation

Table S7 – Details on sedentary behaviour measures per study

Table S8 – Cross-sectional associations with cognition for known associated factors

**Table S1 – Covariate operationalisation**

| **Study** | **HELIAD** | **PATH** | **SALSA** | **SGS** | **SLAS2** |
| --- | --- | --- | --- | --- | --- |
| **Age** | CON – Age centred around 65 | CON – Age centred around 65 | CON – Age centred around 65 | CON – Age centred around 65 | CON – Age centred around 65 |
| **Gender** | BIN – Male/female | BIN – Male/female | BIN – Male/female | BIN – Male/female | BIN – Male/female |
| **Ethnicity** |  |  | BIN – Born foreign yes/no |  | CAT – Ethnicity  1. Chinese  2. Malay  3. Indian  4. Others |
| **Education** | CON - Years of education | CON – Years of education | CON – Years of education | CON – Years of education | CON – Years of education |
| **Income** |  | CAT – Household income  1. No more than $300 p/w  2. More than $300 but no more than $575 p/w  3. More than $575 but no more than $1075 p/w  4. More than $1075 but no more than $1700 p/w  5. More than $1700 but no more than $2400 p/w  6. More than $2400 p/w | CAT – Household month income  1. Less than 1000  2. 1000 to 1499  3. 1500 to 1999  4. 2000 to 2499  5. 2500 or more | CAT – Economic status  1. Very poor  2. Poor  3. Fair  4. Good |  |
| **Marital status** | BIN – Married yes/no | CAT – Current status  1. Married-first and only marriage  2. Remarried-second or later marriage  3. Separated from someone you have been married to  4. Divorced  5. Widowed  6. Have never married | CAT – Marital status  1. Single/never married  2. Married  3. Widowed  4. Divorced  5. Separated  6. Living with someone as a spouse |  | CAT – Marital status  1. Single  2. Married  3. Divorced  4. Widowed |
| **Living status** | CON – Number of people living with | CON – Number of people living with | BIN – Living on your own yes/no | BIN – Living alone or not yes/no | CAT – Living alone, in institution, or with others |
| **BMI** | CON – Weight / height^2^ | CON – Weight / height^2^ | CON – Weight / height^2^ | CON – Weight / height^2^ | CON – Weight / height^2^ |
| **Study (continued).** | **HELIAD** | **PATH** | **SALSA** | **SGS** | **SLAS2** |
| **Smoking** | BIN – Currently  0. No  1. Yes | BIN – Currently  1. Yes  2. No | CAT – Smoking status  1. Never smoked  2. Former smoker  3. Current smoker | CAT – Smoking status  1. Almost every day  2. Sometimes  3. Smoked before but not currently  4. Never | CAT – Smoking status  1. Non-smoker  2. Ex-smoker  3. Current smoker |
| **Alcohol consumption** | BIN – Currently  0. No  1. Yes | CAT – Alcohol frequency  1. Not in the last year  2. Monthly or less  3. 2-3 times a month  4. Once a week  5. 2-3 times a week  6. 4-6 times a week  7. Every day | BIN – Any alcohol consumption  0. No  1. Yes | CAT – Drinking status  1. Almost every day  2. Sometimes  3. Rarely  4. Never | CAT – Alcohol frequency  1. Never or rarely  2. Yes |
| **Physical Activity** | CON – MET hours recreational PA (dancing/walking) | CON – Hours of moderate and vigorous PAs a day | CON – MET hours moderate to vigorous PAs | CON – Fraction of moderate to vigorous PAs time of total awake wear time | CON – Hours of moderate and vigorous PAs a day |
| **Perceived health** |  | CAT – General health  1. Excellent  2. Very good  3. Good  4. Fair  5. Poor | CAT – Health status  1. Excellent  2. Very good  3. Good  4. Fair  5. Poor | CAT – Health status  1. Very good  2. Good  3. Fair  4. Poor | CAT – General health  1. Excellent  2. Very good  3. Good  4. Fair  5. Poor |
| **Morbidities** | CON – Count (of total 24 diseases) | CON – Count (of total 16 diseases) | CON – Count (of total 11 diseases) | CON – Count (of total 8 diseases) | CON – Count (of total 11 diseases) |
| **Blood pressure** | CON – Systolic and diastolic blood pressure | CON – Systolic and diastolic blood pressure | CON – Systolic and diastolic blood pressure |  | CON – Systolic and diastolic blood pressure |
| **Sleep quality** | CON – Sum score 2 items | CON – Sum score 3 items | CON – Sum score 3 items |  | CON – sum score 2 items |
| **Depression** | CON – 15 item GDS sum score | CON – 9 item BPHQ sum score | CON – CESD sum score | CON – K6 sum score | CON – GDS sum score |

*Abbreviations: CON = Continuous variable, BIN = Binary variable, CAT = Categorical variable, p/w = Per week, BMI = Body Mass Index, cig = Cigarettes, PA = Physical Activity, MET = Metabolic Equivalent of Task, GDS = Geriatric Depression Scale, BPHQ = Brief Patient Health Questionnaire, CESD = Center for Epidemiologic Studies Depression scale, K6 = 6 item Kessler Psychological Distress Scale.*

**Figure S1 – Flow chart complete cases**


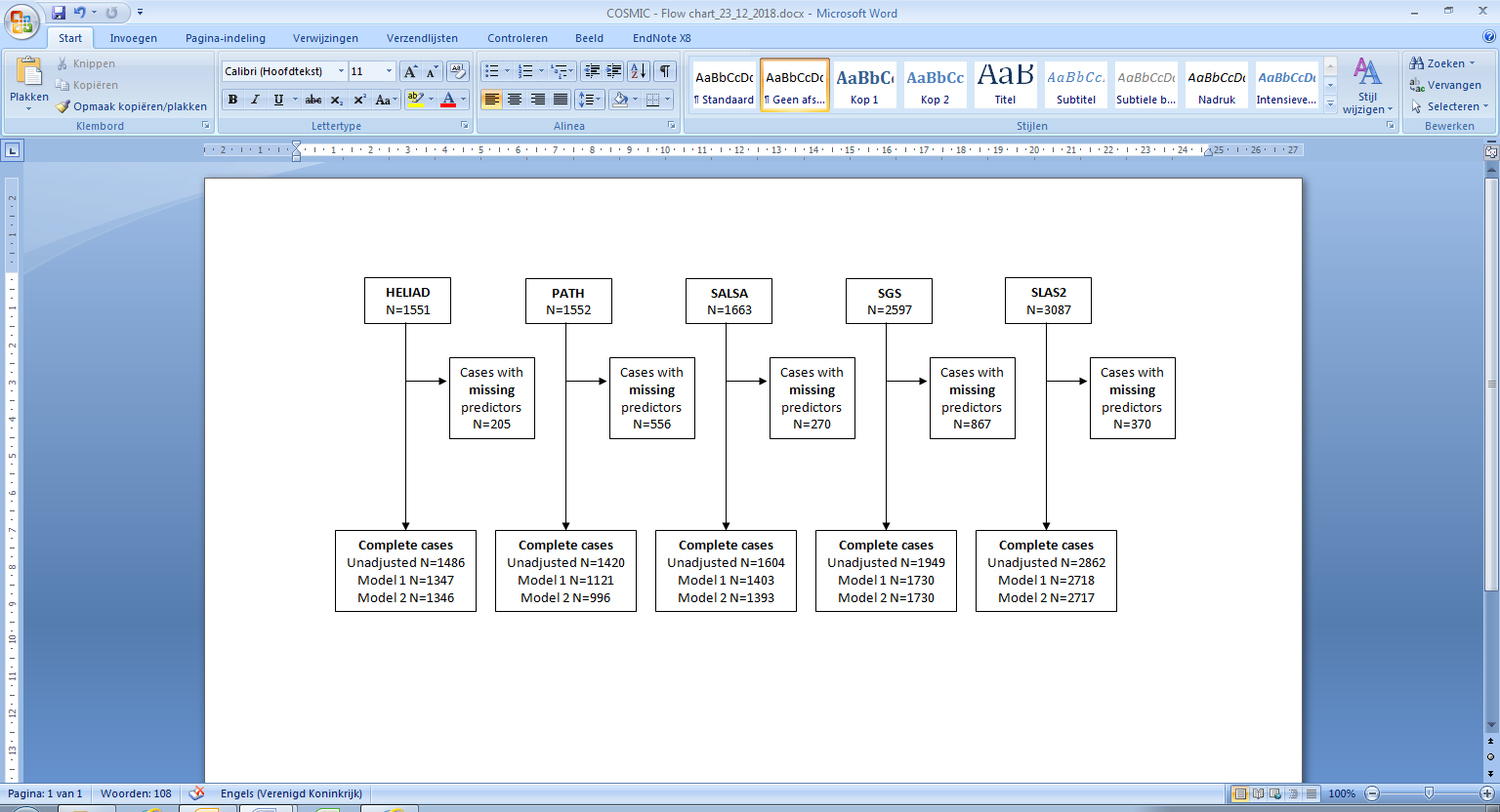


*For the non-imputed datasets, cases may still contain missing values for outcome variables (e.g. MMSE), which will be statistically corrected for when using mixed model analyses. The difference between the original N and unadjusted model N are therefore representative for the amount of participants with missing data on SB.*

**Table S2 – Linear mixed growth model analysis for complete cases on the association of sedentary behaviour on cognition**

| **Study** | **Unadjusted** | | | **Model 1^a^** | | | **Model 2^b^** | | |
| --- | --- | --- | --- | --- | --- | --- | --- | --- | --- |
|  | **B** | **95% CI** | **P-value** | **B** | **95% CI** | **P-value** | **B** | **95% CI** | **P-value** |
| **Cross-sectional effect** | | | | | | | | | |
| **HELIAD** | -0.119 | -0.189 ; -0.049 | <.001 | -0.001 | -0.068 ; 0.066 | .98 | -0.001 | -0.069 ; 0.066 | .97 |
| **PATH** | -0.003 | -0.023 ; 0.017 | .77 | 0.001 | -0.022 ; 0.023 | .94 | -0.005 | -0.029 ; 0.019 | .70 |
| **SALSA^c^** | 0.335 | 0.058 ; 0.612 | .02 | -0.093 | -0.364 ; 0.177 | .50 | -0.066 | -0.340 ; 0.209 | .64 |
| **SGS** | -0.004 | -0.013 ; 0.004 | .33 | -0.001 | -0.011 ; 0.010 | .92 | 0.004 | -0.008 ; 0.015 | .57 |
| **SLAS2** | 0.036 | -0.006 ; 0.077 | .09 | 0.060 | 0.023 ; 0.096 | .001 | 0.110 | 0.070 ; 0.150 | <.001 |
| **Longitudinal effect** | | | | | | | | | |
| **HELIAD** | 0.030 | -0.022 ; 0.081 | .25 | 0.019 | -0.032 ; 0.070 | .46 | 0.019 | -0.032 ; 0.070 | .46 |
| **SALSA^c^** | 0.007 | -0.037 ; 0.051 | .76 | -0.021 | -0.069 ; 0.027 | .40 | -0.023 | -0.072 ; 0.026 | .36 |
| **SGS** | -0.002 | -0.008 ; 0.004 | .49 | 0.000 | -0.007 ; 0.007 | .99 | -0.000 | -0.009 ; 0.009 | .97 |
| **SLAS2** | -0.006 | -0.020 ; 0.007 | .37 | -0.008 | -0.021 ; 0.006 | .28 | -0.009 | -0.024 ; 0.007 | .26 |

*The basic linear mixed model (ignoring covariate adjustment) was parameterized as: Cognition (MMSE or 3MS) = x_1_+ x_2_ × sedentary behaviour + x_3_ × time + x_4_ × time × sedentary behaviour + random intercept for each individual + residual error. The cross sectional effects presented here are then represented in this model as x_2_ and the longitudinal effect is x_4_.*

*^a^ Model 1 is adjusted for age, gender, ethnicity, education, income, alcohol consumption, smoking, BMI, marital status, living status, perceived health, morbidities, blood pressure, sleep quality, depression. In HELIAD not corrected for ethnicity, income, perceived health. In PATH not corrected for ethnicity. In SLAS2 not corrected for income. In SGS not corrected for ethnicity, marital status, blood pressure, sleep quality.*

*^b^ Model 2 is adjusted for all variables of model 1 + PA*

*^c^ SALSA outcome variable is 3MS ranging from 0-100 instead of MMSE ranging from 0-30.*

**Table S3 – Linear mixed model analysis on the cross-sectional association of different types of sedentary behaviour on cognition in SALSA**

| **Study** | **Mean (SE) h/day** | **Unadjusted** | | | **Model 1^a^** | | | **Model 2^b^** | | |
| --- | --- | --- | --- | --- | --- | --- | --- | --- | --- | --- |
|  |  | **B** | **95% CI** | **P-value** | **B** | **95% CI** | **P-value** | **B** | **95% CI** | **P-value** |
| **Complete cases** | | | | | | | | | | |
| **Home** | 3.6 (0.05) | -0.070 | -0.118 ; -0.021 | .005 | -0.037 | -0.085 ; 0.012 | .14 | -0.034 | -0.083 ; 0.016 | .18 |
| **Work** | 0.3 (0.03) | 0.122 | 0.038 ; 0.205 | .004 | -0.035 | -0.117 ; 0.046 | .39 | -0.027 | -0.110 ; 0.056 | .53 |
| **Drive** | 0.7 (0.03) | 0.298 | 0.215 ; 0.382 | <.001 | 0.082 | -0.005 ; 0.168 | .06 | 0.081 | -0.006 ; 0.168 | .07 |
| **Imputed cases** | | | | | | | | | | |
| **Home** | 3.6 (0.05) | -0.080 | -0.134 ; -0.026 | .004 | -0.033 | -0.083 ; 0.017 | .19 | -0.029 | -0.080 ; 0.021 | .25 |
| **Work** | 0.3 (0.03) | 0.129 | 0.043 ; 0.216 | .003 | -0.039 | -0.119 ; 0.040 | .34 | -0.034 | -0.114 ; 0.046 | .41 |
| **Drive** | 0.7 (0.03) | 0.323 | 0.236 ; 0.409 | <.001 | 0.097 | 0.013 ; 0.181 | .02 | 0.095 | 0.011 ; 0.179 | .03 |

*^a^ Model 1 is adjusted for age, gender, ethnicity, education, income, alcohol consumption, smoking, BMI, marital status, living status, perceived health, morbidities, blood pressure, sleep quality, depression.*

*^b^ Model 2 is adjusted for all variables of model 1 + PA*

**Table S4 – Linear mixed growth model analysis for imputed cases on the association of sedentary behaviour on cognition stratified by physical activity level**

|  |  | **Unadjusted** | | | **Model 1^a^** | | |
| --- | --- | --- | --- | --- | --- | --- | --- |
|  | **PA** | **B** | **95% CI** | **P-value** | **B** | **95% CI** | **P-value** |
| **Cross-sectional effect** | | | | | | | |
| **HELIAD** | - | -0.121 | -0.229 ; -0.012 | 0.03 | -0.045 | -0.146 ; 0.057 | 0.39 |
|  | + | -0.118 | -0.200 ; -0.035 | 0.01 | -0.008 | -0.085 ; 0.069 | 0.84 |
| **PATH** | - | -0.005 | -0.032 ; 0.023 | 0.74 | 0.000 | -0.028 ; 0.028 | 0.98 |
|  | + | 0.007 | -0.031 ; 0.044 | 0.73 | 0.111 | -0.025 ; 0.048 | 0.55 |
| **SALSA^b^** | - | -0.043 | -0.473 ; 0.386 | 0.84 | -0.420 | -0.805 ; -0.035 | 0.03 |
|  | + | 0.931 | 0.506 ; 1.356 | <0.001 | 0.457 | 0.063 ; 0.852 | 0.02 |
| **SGS** | - | 0.002 | -0.015 ; 0.019 | 0.78 | 0.005 | -0.013 ; 0.023 | 0.60 |
|  | + | 0.011 | -0.002 ; 0.024 | 0.10 | 0.007 | -0.007 ; 0.020 | 0.34 |
| **SLAS2** | - | 0.026 | -0.037 ; 0.088 | 0.42 | 0.046 | -0.008 ; 0.100 | 0.10 |
|  | + | 0.160 | 0.087 ; 0.232 | <0.001 | 0.186 | 0.123 ; 0.249 | <0.001 |
| **Longitudinal effect** | | | | | | | |
| **HELIAD** | - | 0.054 | -0.016 ; 0.123 | 0.13 | 0.072 | 0.003 ; 0.141 | 0.04 |
|  | + | -0.012 | -0.085 ; 0.060 | 0.73 | -0.053 | -0.123 ; 0.018 | 0.14 |
| **SALSA^b^** | - | 0.003 | -0.062 ; 0.068 | 0.93 | 0.010 | -0.057 ; 0.078 | 0.76 |
|  | + | 0.002 | -0.063 ; 0.067 | 0.95 | -0.033 | -0.101 ; 0.035 | 0.34 |
| **SGS** | - | -0.003 | -0.014 ; 0.009 | 0.67 | -0.001 | -0.014 ; 0.012 | 0.85 |
|  | + | -0.002 | -0.011 ; 0.006 | 0.60 | 0.001 | -0.009 ; 0.010 | 0.88 |
| **SLAS2** | - | -0.013 | -0.034 ; 0.008 | 0.22 | -0.017 | -0.012 ; 0.001 | 0.11 |
|  | + | -0.003 | -0.025 ; 0.019 | 0.79 | -0.003 | -0.025 ; 0.018 | 0.77 |

*The basic linear mixed model (ignoring covariate adjustment) was parameterized as: Cognition (MMSE or 3MS) = x_1_+ x_2_ × sedentary behaviour + x_3_ × time + x_4_ × time × sedentary behaviour + random intercept for each individual + residual error. The cross sectional effects presented here are then represented in this model as x_2_ and the longitudinal effect is x_4_. The analysis was stratified by PA which was categorised based on median PA ( - =low, + = high PA).*

*^a^ Model 1 is adjusted for age, gender, ethnicity, education, income, alcohol consumption, smoking, BMI, marital status, living status, perceived health, morbidities, blood pressure, sleep quality, depression. In HELIAD not corrected for ethnicity, income, perceived health. In PATH not corrected for ethnicity. In SLAS2 not corrected for income. In SGS not corrected for ethnicity, marital status, blood pressure, sleep quality.*

*^b^ SALSA outcome variable is 3MS ranging from 0-100 instead of MMSE ranging from 0-30.*

**Table S5 – Linear mixed growth model analysis for imputed cases on the association of sedentary behaviour on cognition stratified by gender**

|  |  | **Unadjusted** | | | **Model 1^a^** | | | **Model 2^b^** | | |
| --- | --- | --- | --- | --- | --- | --- | --- | --- | --- | --- |
|  | **Gender** | **B** | **95% CI** | **P-value** | **B** | **95% CI** | **P-value** | **B** | **95% CI** | **P-value** |
| **Cross-sectional effect** | | | | | | | | | | |
| **HELIAD** | M | -0.105 | -0.206 ; -0.004 | 0.04 | -0.019 | -0.116 ; 0.078 | 0.71 | -0.019 | -0.116 ; 0.078 | 0.70 |
|  | F | -0.127 | -0.220 ; -0.035 | 0.01 | -0.038 | -0.123 ; 0.047 | 0.38 | -0.037 | -0.122 ; 0.048 | 0.39 |
| **PATH** | M | 0.011 | -0.019 ; 0.041 | 0.46 | 0.016 | -0.014 ; 0.046 | 0.30 | 0.012 | -0.019 ; 0.042 | 0.45 |
|  | F | -0.012 | -0.040 ; 0.017 | 0.42 | -0.011 | -0.041 ; 0.018 | 0.45 | -0.009 | -0.039 ; 0.021 | 0.55 |
| **SALSA^c^** | M | 0.160 | -0.266 ; 0.585 | 0.46 | -0.108 | -0.494 ; 0.277 | 0.58 | -0.089 | -0.481 ; 0.303 | 0.66 |
|  | F | 0.418 | -0.040 ; 0.877 | 0.07 | -0.061 | -0.466 ; 0.344 | 0.77 | -0.034 | -0.440 ; 0.372 | 0.87 |
| **SGS** | M | 0.004 | -0.010 ; 0.018 | 0.55 | 0.008 | -0.007 ; 0.022 | 0.30 | 0.018 | 0.001 ; 0.035 | 0.04 |
|  | F | -0.018 | -0.032 ; -0.005 | 0.01 | -0.002 | -0.016 ; 0.012 | 0.73 | -0.004 | -0.021 ; 0.013 | 0.66 |
| **SLAS2** | M | 0.062 | 0.006 ; 0.119 | 0.03 | 0.075 | 0.023 ; 0.127 | 0.005 | 0.100 | 0.045 ; 0.156 | <0.001 |
|  | F | 0.001 | -0.062 ; 0.064 | 0.98 | 0.057 | 0.003 ; 0.112 | 0.04 | 0.138 | 0.076 ; 0.200 | <0.001 |
| **Longitudinal effect** | | | | | | | | | | |
| **HELIAD** | M | 0.036 | -0.039 ; 0.110 | 0.35 | 0.015 | -0.060 ; 0.091 | 0.70 | 0.016 | -0.059 ; 0.092 | 0.67 |
|  | F | 0.028 | -0.039 ; 0.096 | 0.41 | 0.040 | -0.023 ; 0.104 | 0.21 | 0.041 | -0.023 ; 0.104 | 0.21 |
| **SALSA^c^** | M | -0.004 | -0.071 ; 0.064 | 0.92 | -0.011 | -0.079 ; 0.059 | 0.77 | -0.011 | -0.081 ; 0.058 | 0.75 |
|  | F | 0.010 | -0.053 ; 0.074 | 0.75 | 0.004 | -0.063 ; 0.071 | 0.91 | -0.001 | -0.068 ; 0.065 | 0.97 |
| **SGS** | M | -0.006 | -0.016 ; 0.005 | 0.29 | -0.006 | -0.017 ; 0.005 | 0.26 | -0.009 | -0.022 ; 0.003 | 0.14 |
|  | F | 0.003 | -0.005 ; 0.012 | 0.44 | 0.003 | -0.006 ; 0.013 | 0.49 | 0.006 | -0.005 ; 0.018 | 0.27 |
| **SLAS2** | M | -0.011 | -0.027 ; 0.007 | 0.23 | -0.012 | -0.029 ; 0.005 | 0.17 | -0.011 | -0.030 ; 0.008 | 0.28 |
|  | F | -0.007 | -0.027 ; 0.013 | 0.47 | -0.010 | -0.029 ; 0.010 | 0.33 | -0.013 | -0.035 ; 0.009 | 0.26 |

*The basic linear mixed model (ignoring covariate adjustment) was parameterized as: Cognition (MMSE or 3MS) = x_1_+ x_2_ × sedentary behaviour + x_3_ × time + x_4_ × time × sedentary behaviour + random intercept for each individual + residual error. The cross sectional effects presented here are then represented in this model as x_2_ and the longitudinal effect is x_4_. The analysis was stratified by gender (1=male, 2=female).*

*^a^ Model 1 is adjusted for age, gender, ethnicity, education, income, alcohol consumption, smoking, BMI, marital status, living status, perceived health, morbidities, blood pressure, sleep quality, depression. In HELIAD not corrected for ethnicity, income, perceived health. In PATH not corrected for ethnicity. In SLAS2 not corrected for income. In SGS not corrected for ethnicity, marital status, blood pressure, sleep quality.*

*^b^ Model 2 is adjusted for all variables of model 1 + PA*

*^c^ SALSA outcome variable is 3MS ranging from 0-100 instead of MMSE ranging from 0-30.*

**Table S6 – Correlations of sedentary behaviour with imputed covariables**

|  | **HELIAD** | | **PATH** | | **SALSA**^a^ | | **SGS** | | **SLAS2** | |
| --- | --- | --- | --- | --- | --- | --- | --- | --- | --- | --- |
|  | **Corr** | **P-value** | **Corr** | **P-value** | **Corr** | **P-value** | **Corr** | **P-value** | **Corr** | **P-value** |
| **Gender^b^** | 0.030 | 0.26 | -0.048 | 0.06 | -0.191 | <0.001 | -0.327 | <0.001 | -0.091 | <0.001 |
| **Age** | 0.058 | .03 | -0.023 | .38 | -0.022 | .38 | 0.234 | <.001 | 0.116 | <.001 |
| **Education** | -0.186 | <.001 | 0.049 | .06 | 0.155 | <.001 | 0.050 | .02 | -0.002 | .90 |
| **Income** |  |  | 0.046^d^ | .09 | 0.157^d^ | <.001 | 0.044^d^ | .05 |  |  |
| **Alcohol consumption** | -0.040^d^ | .12 | -0.049^d^ | .05 | 0.039^d^ | .12 | 0.056^c,d^ | .02 | 0.025^d^ | .19 |
| **Smoking** | 0.050^d^ | .05 | 0.010^c,d^ | .69 | 0.120^d^ | <.001 | 0.257^c,d^ | <.001 | 0.080^d^ | <.001 |
| **BMI** | 0.077 | .003 | 0.223 | <.001 | 0.035 | .18 | 0.096 | <.001 | 0.060 | .002 |
| **Perceived health** |  |  | -0.124^c,d^ | <.001 | 0.022^c,d^ | .38 | -0.167^c,d^ | <.001 | -0.066^c,d^ | <.001 |
| **Morbidities** | 0.111 | <.001 | 0.105 | <.001 | 0.051 | .04 | 0.132 | <.001 | 0.121 | <.001 |
| **SBP** | 0.045 | .09 | -0.054 | .05 | -0.023 | .37 |  |  | 0.012 | .53 |
| **Sleep quality** | -0.090 | <.001 | -0.051^c^ | .05 | 0.019 | .46 |  |  | -0.017^c^ | .36 |
| **Depression** | 0.031 | .24 | 0.127 | <.001 | -0.004 | .86 | 0.020 | .38 | 0.025 | .21 |
| **PA** | -0.055 | .04 | -0.155 | <.001 | -0.097 | <.001 | -0.565 | <.001 | -0.463 | <.001 |

*Correlations shown are the ones represented in Figure 1.*

*^a^ SALSA outcome variable is 3MS ranging from 0-100 instead of MMSE ranging from 0-30.*

*^b^ Coded 1=male, 2=female*

*^c^ Reversely coded*

*^d^ Spearman correlations instead of Pearson correlations*

**S1 Results - Model evaluation**

The correlation analysis with three known associated factors, e.g. BMI, age, and morbidity count, was used to evaluate the associations of the SB measures with these factors, shown to be associated with SB in prior studies. Positive significant but weak correlations between SB and BMI, age, and morbidity count were found in respectively four, three, and five studies (Fig. 1, and Table S6). SB measures were further evaluated by assessing the measures on ceiling and floor effects, inter-quartile range, and medians (see Table S7). No strong ceiling and floor effects were found in all of the studies.

To further evaluate the model used, three known associated (risk) factors, already included in the model as confounders, were analysed for their cross-sectional association in the fully adjusted models for MMSE/3MS scores. Age and depression were negatively associated, and education was positively associated, in all studies with cognition (Table S8).

**Table S7 – Details on sedentary behaviour measures per study**

|  | **HELIAD** | **PATH** | **SALSA** | **SGS** | **SLAS2** |
| --- | --- | --- | --- | --- | --- |
| **N** | 1486 | 1530 | 1604 | 1949 | 2862 |
| **Mean^a^** | 3.5 | 7.1 | 4.6 | 7.4 | 6.1 |
| **SD** | 2.1 | 2.7 | 2.3 | 2.1 | 2.3 |
| **Range** | 20.0 | 23.0 | 16.0 | 17.3 | 17.1 |
| **Median** | 3.0 | 6.6 | 4.1 | 7.4 | 6.0 |
| **Q1-Q3** | 2.0 – 5.0 | 5.0 – 8.3 | 3.0 – 6.0 | 6.1 – 8.7 | 4.6 – 7.4 |
| **Missings N (%)** | 65 (4.2) | 22 (1.4) | 59 (3.6) | 648 (25.0) | 225 (7.3) |

*^a^ Hours per day*

**Table S8 – Cross-sectional associations with cognition for known associated factors**

| **Study** | **Age** | | | **Education** | | | **Depression** | | |
| --- | --- | --- | --- | --- | --- | --- | --- | --- | --- |
|  | **B** | **95% CI** | **P-value** | **B** | **95% CI** | **P-value** | **B** | **95% CI** | **P-value** |
| **Complete cases** | | | | | | | | | |
| **HELIAD** | -0.079 | -0.105 ; -0.052 | <.001 | 0.200 | 0.166 ; 0.233 | <.001 | -0.075 | -0.119 ; -0.031 | <.001 |
| **PATH** | -0.070 | -0.111 ; -0.030 | <.001 | 0.075 | 0.049 ; 0.101 | <.001 | -0.034 | -0.059 ; -0.010 | .006 |
| **SALSA^a^** | -0.183 | -0.286 ; -0.079 | <.001 | 0.826 | 0.679 ; 0.972 | <.001 | -0.091 | -0.156 ; -0.025 | .007 |
| **SGS** | -0.050 | -0.073 ; -0.027 | <.001 | 0.223 | 0.171 ; 0.274 | <.001 | -0.065 | -0.101 ; -0.029 | <.001 |
| **SLAS2** | -0.071 | -0.084 ; -0.058 | <.001 | 0.187 | 0.166 ; 0.208 | <.001 | -0.196 | -0.256 ; -0.136 | <.001 |
| **Imputed cases** | | | | | | | | | |
| **HELIAD** | -0.074 | -0.099 ; -0.050 | <.001 | 0.190 | 0.159 ; 0.221 | <.001 | -0.079 | -0.121 ; -0.038 | <.001 |
| **PATH** | -0.048 | -0.084 ; -0.012 | .010 | 0.072 | 0.049 ; 0.096 | <.001 | -0.024 | -0.045 ; -0.003 | .03 |
| **SALSA^a^** | -0.275 | -0.371 ; -0.180 | <.001 | 0.865 | 0.725 ; 1.005 | <.001 | -0.099 | -0.167 ; -0.032 | .004 |
| **SGS** | -0.064 | -0.086 ; -0.043 | <.001 | 0.248 | 0.200 ; 0.295 | <.001 | -0.060 | -0.094 ; -0.026 | <.001 |
| **SLAS2** | -0.073 | -0.086 ; -0.060 | <.001 | 0.198 | 0.176 ; 0.219 | <.001 | -0.192 | -0.249 ; -0.136 | <.001 |

*All estimates from model 2, reported in table 3: adjusted for age, gender, ethnicity, education, income, alcohol consumption, smoking, BMI, marital status, living status, perceived health, morbidities, blood pressure, sleep quality, depression, PA, and SB. In HELIAD not corrected for ethnicity, income, perceived health. In PATH not corrected for ethnicity. In SLAS2 not corrected for income. In SGS not corrected for ethnicity, marital status, blood pressure, sleep quality.*

*^a^ SALSA outcome variable is 3MS ranging from 0-100 instead of MMSE ranging from 0-30.*
